# The Effects on the Growth of HIV-exposed Uninfected Infants of Initiating Dolutegravir-based Versus Efavirenz-based cART in Late Pregnancy (DolPHIN-2)

**DOI:** 10.1097/INF.0000000000004902

**Published:** 2025-07-18

**Authors:** Lisa Hagens, Lisanne A. H. Bevers, Thokozile R. Malaba, Sylvia Cornelia Nassiwa, Megan Mrubata, Helene Theunissen, Helen Reynolds, Nengjie He, Jim Read, David M. Burger, Mohammed Lamorde, Landon Myer, Duolao Wang, Saye Khoo, Catriona Waitt, Angela Colbers

**Affiliations:** From the * Department of Pharmacy, Research Institute for Medical Innovation, Radboud University Medical Center, Nijmegen, The Netherlands; † Division of Epidemiology & Biostatistics, School of Public Health, University of Cape Town, Cape Town, South Africa; ‡ Research Department, Infectious Diseases Institute, Makerere University, Kampala, Uganda; § Department of Pharmacology & Therapeutics, University of Liverpool, Liverpool, United Kingdom; ¶ Department of Clinical Sciences, Liverpool School of Tropical Medicine, Liverpool, United Kingdom.

**Keywords:** HIV, antiretroviral therapy, infant growth and pregnancy

## Abstract

**Background:**

In 2019, the World Health Organization (WHO) changed its recommendations for pregnant women living with HIV from efavirenz-based to dolutegravir-based therapy due to its superior efficacy, tolerability and resistance profile. Perinatal exposure to antiretrovirals may influence infant growth, but limited data exist on the effects of specific regimens over time.

**Aim:**

This study aimed to compare growth trajectories over the first 72 weeks of life among infants exposed to dolutegravir-based versus efavirenz-based therapy during late pregnancy.

**Methods:**

The DolPHIN-2 trial was a randomized, open-label trial conducted in South Africa and Uganda, researching the efficacy of dolutegravir-based versus efavirenz-based therapy in pregnant women living with HIV, initiating treatment in the third trimester. In this secondary analysis, we compared growth trajectories until 72 weeks postpartum between HIV-exposed uninfected infants perinatally exposed to dolutegravir-based versus efavirenz-based therapy. Measures of infant weight, length and head circumference were converted to WHO-defined weight-for-age, weight-for-length, length-for-age and head circumference-for-age Z-scores. Subsequently, Z-scores were compared across treatment arms, using linear mixed-effect models.

**Results:**

After exclusions, 232 infants remained (dolutegravir: n = 116; efavirenz: n = 116). In both crude models and models adjusted for study site and maternal height, length-for-age Z-scores were 0.277 units higher in the dolutegravir arm. No statistically significant impact of treatment was observed for other outcomes. In both study arms, a decline in mean length-for-age Z-scores occurred over the first 72 weeks, while mean weight-for-age Z-scores declined between weeks 48 and 72.

**Conclusion:**

Our data support the WHO in recommending dolutegravir-based therapy over efavirenz-based therapy in pregnant women living with HIV.

Annually, approximately 1.3 million women living with HIV give birth.^1^ The increasing use of antiretroviral therapy (ART) during pregnancy has significantly decreased the risk of mother-to-child transmission.^2^ In 2019, the World Health Organization (WHO) updated its guidelines on ART, recommending a dolutegravir-based regimen instead of an efavirenz-based regimen for the first- and second-line treatment of all individuals with HIV, including women who are pregnant or of childbearing potential.^3^ This change was supported by the superior efficacy, tolerability and resistance profile of dolutegravir-based compared with efavirenz-based therapy in pregnant women.^4^

While the use of ART during pregnancy offers significant benefits for both mother and child, several studies have shown that perinatal ART exposure might affect fetal, infant and child growth.^5–9^ Previous research has mainly focused on infant growth outcomes at birth, but regimen-specific randomized trials with extended follow-up are lacking.

The Virologic Efficacy and Safety of ART Combinations with TAF/TDF, EFV, and DTG (VESTED) trial is the only study that has compared the effects of perinatal exposure to specific ART regimens on infant growth over time. Within this trial, conducted in 9 countries (Botswana, India, Brazil, South Africa, Tanzania, Uganda, Thailand, the USA and Zimbabwe), growth patterns were compared between infants exposed to dolutegravir-based versus efavirenz-based ART during the second and third trimester of pregnancy and breastfeeding. Infants in the dolutegravir arm presented with lower rates of low birth weight (<2500 g) and stunting [length-for-age Z-score (LAZ) less than −2 SD] compared with those in the efavirenz arm. Additionally, infants in the dolutegravir arm exhibited healthier weight-for-age Z-scores (WAZ) and LAZ at weeks 26 and 50 of life compared with those in the efavirenz arm.^10^ Thus, infants with perinatal exposure to dolutegravir-based regimens showed more optimal growth patterns compared with those with perinatal exposure to efavirenz-based regimens. Healthier infant growth patterns are associated with lower risks of childhood morbidity and mortality and improved cognitive and motor development.^11^

The Dolutegravir in Late‑Presenter HIV‑Positive Pregnant Women (DolPHIN-2) trial compared growth trajectories over the first 72 weeks of life in infants whose mothers initiated dolutegravir-based versus efavirenz-based ART in the third trimester. This contrasts with the VESTED trial, which reported growth outcomes up to only 50 weeks in infants born to women who initiated ART in the second trimester. Research on ART initiation in the third trimester is of great importance, as in many settings where HIV is prevalent, women often present late to antenatal care, resulting in HIV diagnosis later in pregnancy and consequently delayed initiation of treatment.^12^ Here, we compared growth trajectories over the first 72 weeks of life in infants exposed to dolutegravir-based versus efavirenz-based ART during late pregnancy and breastfeeding.

## MATERIALS AND METHODS

### Study Design and Participants

The DolPHIN-2 trial was a randomized, open-label trial performed in South Africa and Uganda evaluating ART initiation in women diagnosed with HIV (age ≥18 years) in the third trimester of pregnancy (NCT03249181). The design and primary outcomes of this trial have been described elsewhere.^4^

In this prespecified secondary analysis, we compared growth trajectories over the first 72 weeks of life in HIV-exposed uninfected infants exposed to dolutegravir-based versus efavirenz-based ART during late pregnancy (≥28 weeks) and breastfeeding.

### Ethical Approval and Informed Consent

Ethical approval was obtained from research ethics committees located in the United Kingdom, South Africa and Uganda. All participating mothers provided written informed consent at enrollment and again for continued participation of themselves and their child in the trial after giving birth.

### Randomization and Blinding

The participating women were randomized 1:1, using block randomization with a block size of 4, stratified by country. In total, 133 women were randomized to receive efavirenz 600 mg daily, and 135 participants were randomized to receive dolutegravir 50 mg daily. Treatment was combined with 300 mg tenofovir disoproxil fumarate together with either 300 mg lamivudine (Uganda) or 200 mg emtricitabine (South Africa) taken orally once daily.

### Procedures

Gestational age was determined at screening using a combination of symphysis-fundal height, fetal ultrasound, and/or the last menstrual period. Clinical assessment was performed around delivery (+14 days) and on all postpartum visits (scheduled around weeks 6, 12, 24, 48 and 72). During this assessment, neonatal length, weight and head circumference were measured, as well as maternal weight. Maternal height was determined once at screening. All measurements were performed according to local standard operating procedures. During each follow-up visit, mothers were asked to report whether the infant was exclusively breastfed, partially breastfed or not breastfed. Follow-up of mothers and infants continued until at least 72 weeks postpartum.

### Outcomes

The primary outcomes of this study were the differences in WAZ, weight-for-length Z-scores (WLZ), LAZ and head circumference-for-age Z-scores (HCZ) between treatment arms over the first 72 weeks of life. Infant growth outcomes were transformed into Z-scores to account for variations in sex, and either age (for WAZ, LAZ and HCZ) or length (for WLZ). Z-scores for preterm infants (gestational age <37 weeks) were calculated using the 2013 Fenton preterm growth charts, specifically designed to assess the growth patterns of preterm infants until a postmenstrual age of 50 weeks.^13^ Z-scores for term infants (gestational age ≥37 weeks) and for preterm infants after 50 weeks of postmenstrual age were calculated using WHO Anthro software V3.2.2 based on global WHO-defined growth charts. Biologically implausible Z-scores were excluded according to WHO guidelines.^14^ When multiple measurements were available within the same week, only the first measure was used.

Twins were excluded from the analyses because of their potential for fetal growth restriction.^15^ Additionally, one early neonatal death was excluded due to limited data. Furthermore, infants who acquired HIV were excluded because of the negative impact of HIV on infant growth.^16^

### Statistical Analysis

Mean WAZ, WLZ, LAZ and HCZ, along with their 95% confidence intervals (CIs), were determined at weeks 0, 6 (±2 weeks), 12 (±2 weeks), 24 (±4 weeks), 48 (±4 weeks) and 72 (±4 weeks). Results were computed using GraphPad Prism V9.5.0.

Linear mixed-effect models were used to compare WAZ, WLZ, LAZ and HCZ between treatment arms over time. Infant IDs were included as subject to account for the correlation between multiple measurements within the same individual over time, with a random intercept. A random slope on time was used to allow the effect of time to vary between individuals.

First, the models were evaluated for significant differences in slopes, either continuous or categorical (weeks 0–6, 6–12, 12–24, 24–48 and 48–72), between treatment arms by adding an interaction term for week category by treatment. When no statistically significant differences in slopes were identified, the average difference in the height of the curve was compared between treatments. All models were adjusted for study site when adjustments improved model fit, as determined by the Akaike information criterion (AIC), and the WLZ model was additionally adjusted for maternal height when this adjustment improved model fit, given the established influence of these factors on infant growth.^17,18^ Parameter coefficients were estimated using the restricted maximum likelihood method in IBM SPSS Statistics 29, with statistical significance set at *P* <0.05.

## RESULTS

### Baseline Characteristics

Anthropometric data were available from 240 infants. Twins (n = 4), early neonatal deaths (n = 1) and infants with congenital HIV infection (n = 3) were excluded from the analyses, leaving 232 infants. One infant in the efavirenz arm was diagnosed with HIV at the last visit (week 72) having previously tested negative for HIV. Due to the late diagnosis, this infant was included in all analyses. 35% of participants did not complete the 72 weeks of follow-up.

Characteristics of women (in the third trimester) and infants are presented in Table 1. Each study arm had 116 participants. Median maternal age, height and gestational age at baseline were similar in the study arms. Women in the dolutegravir arm had a higher median baseline weight (75 vs. 69 kg) and body mass index (30 vs. 27 kg/m^2^) than women in the efavirenz arm. Overall, more boys were included than girls (126 vs. 106), equally distributed between treatment arms. Median gestational age at birth was similar between study arms. The average duration of exclusive breastfeeding was longer in the dolutegravir arm compared with that in the efavirenz arm (3 months vs. 1 month). The number of preterm births was comparable between treatment arms (dolutegravir: n = 20; efavirenz: n = 18).

**TABLE 1.** Maternal and Infant Baseline Characteristics by Regimen

	Dolutegravir	Efavirenz	Total
Total, n (%)	116 (50%)	116 (51%))	232 (100%)
Maternal characteristics (measured in the third trimester at baseline)			
Age, years, n (%)			
≤24	34 (29%)	34 (29%)	68 (29%)
25–29	33 (28%)	44 (38%)	77 (33%)
≥30	49 (42%)	38 (33%)	87 (38%)
Median (IQR)	28 (24–32)	28 (24–31)	28 (24–32)
Gestational age at baseline in weeks, median (IQR)	31 (29–34)	31 (29–34)	31 (29–34)
Weight, kg, median (IQR)	75 (65–86)	69 (61–82)	72 (62–83)
Missing data, n (%)	1 (0.9%)	0 (0%)	1 (0.4%)
Height, cm, median (IQR)	158 (153–162)	158 (153–162)	158 (153–162)
Missing data, n (%)	1 (0.9%)	0 (0%)	1 (0.4%)
BMI, kg/m^2^, n (%)			
<18.5	0 (0%)	0 (0%)	0 (0%)
18.5–24.9	26 (22%)	31 (27%)	57 (25%)
25.0–29.9	30 (26%)	42 (36%)	72 (31%)
≥30.0	59 (51%)	43 (37%)	102 (44%)
Missing data	1 (0.9%)	0 (0%)	1 (0.4%)
Median (IQR)	30 (25–35)	27 (25–31)	28 (25–33)
Married, n (%)	14 (12%)	11 (10%)	25 (11%)
Missing data	27 (23%)	32 (28%)	59 (25%)
Employed, n (%)	37 (32%)	30 (26%)	67 (29%)
Missing data	27 (23%)	32 (28%)	59 (25%)
Highest education, n (%)			
None	5 (4%)	1 (0.9%)	6 (2.6%)
Primary	15 (13%)	22 (19%)	37 (16%)
Secondary	64 (55%)	55 (47%)	119 (51%)
Tertiary/Vocational	2 (2%)	3 (3%)	5 (2%)
Higher/University	3 (3%)	3 (3%)	6 (3%)
Missing data	27 (23%)	32 (28%)	59 (25%)
Infant characteristics			
Sex, n (%)			
Girl	54 (47%)	52 (45%)	106 (46%)
Boy	62 (53%)	64 (55%)	126 (54%)
Study site, n (%)			
Uganda	58 (50%)	60 (52%)	118 (51%)
South Africa	58 (50%)	56 (48%)	114 (49%)
Preterm births (%)	20 (17%)	18 (16%)	38 (17%)
Gestational age at birth in weeks, median (IQR)	39 (37–41)	39 (38–41)	39 (38–41)
Exclusive breastfeeding, n (%)			
<3 months	66 (57%)	75 (65%)	141 (61%)
3–6 months	31 (27%)	32 (28%)	63 (27%)
≥6 months	17 (15%)	5 (4%)	22 (10%)
Missing data	2 (2%)	4 (3%)	6 (3%)
Duration of exclusive breastfeeding, months, median (IQR)*	3 (1–6)	1 (1–3)	3 (0–6)

* Missing data excluded.

BMI indicates body mass index; IQR, interquartile range; n, number of infants.

### Comparison of Infant Growth Patterns Between Study Arms

**FIGURE 1.**
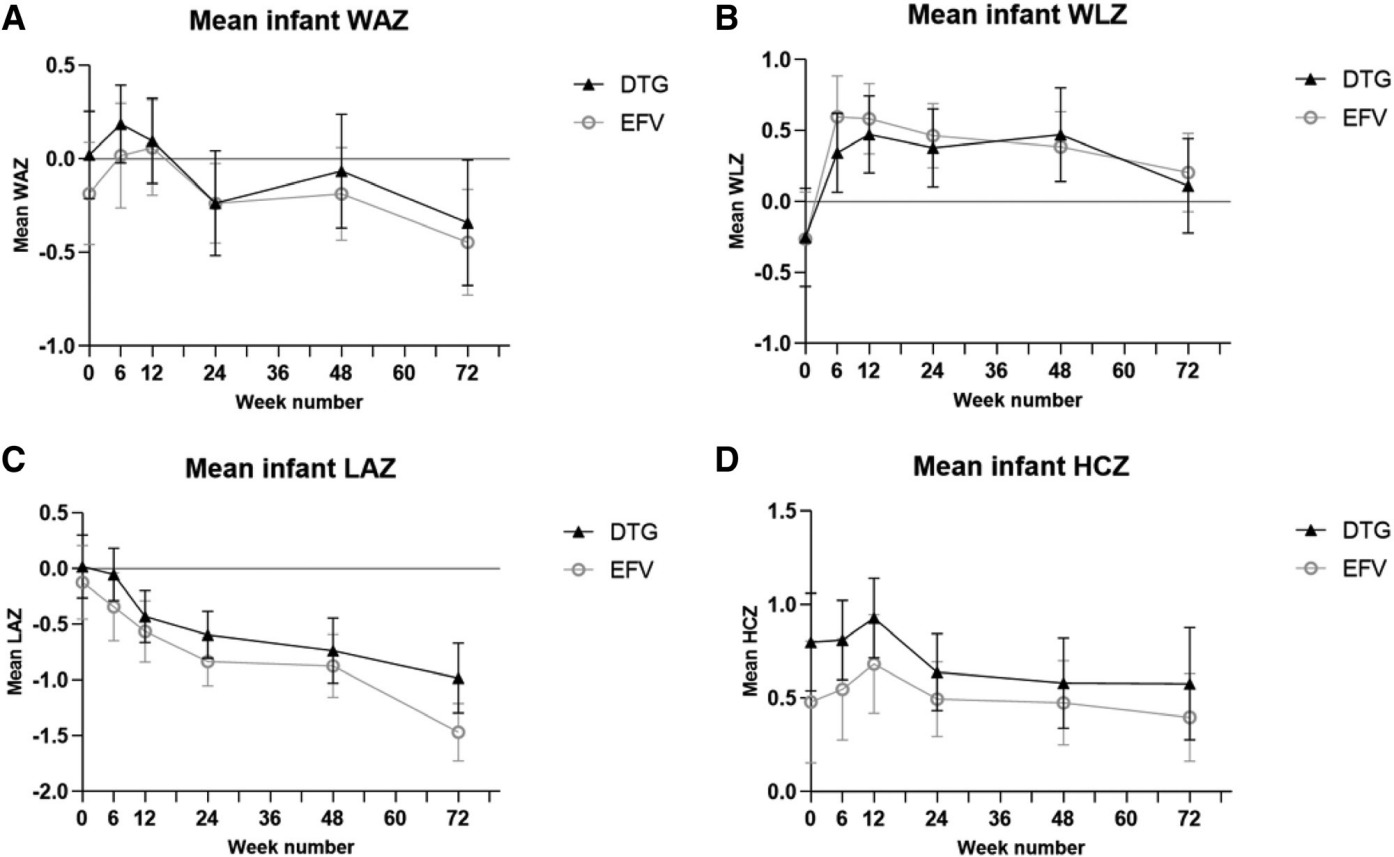
Mean weight-for-age Z-scores (WAZ) (A), weight-for-length Z-scores (WLZ) (B), length-for-age Z-scores (LAZ) (C) and head circumference-for-age Z-scores (HCZ) (D), with 95% confidence intervals, over the first 72 weeks of life. Results are stratified by treatment arm: dolutegravir (DTG; filled triangle) or efavirenz (EFV; open circle). Means are depicted at study visits around weeks 0, 6 (±2 weeks), 12 (±2 weeks), 24 (±4 weeks), 48 (±4 weeks) and 72 (±4 weeks). Results outside of these intervals are excluded from the figure.

Figure 1 shows infant growth trajectories over the first 72 weeks of life by treatment arm. At all study visits, mean LAZ were closer to WHO standards (LAZ = 0) in the dolutegravir arm, while mean HCZ were closer to these standards in the efavirenz arm. No notable differences were observed in mean WAZ and WLZ between treatment arms. For all outcomes, the 95% CIs demonstrated no significant differences between treatment arms over follow-up. Mean WAZ decreased between 48 and 72 weeks of life, and mean LAZ decreased substantially over the first 72 weeks of life. Infant growth trajectories over the first 72 weeks of life by treatment for preterm and term infants separately are shown in Figure, Supplemental Digital Content 1, https://links.lww.com/INF/G279 and 2, https://links.lww.com/INF/G280.

No significant differences in continuous or categorical slopes were observed between treatment arms, as shown in Tables, Supplemental Digital Content 3, https://links.lww.com/INF/G281 and 4, https://links.lww.com/INF/G282. Therefore, the average difference in the height of the curve was compared between treatments.

Table 2 presents the crude and adjusted linear mixed-effect models used in the repeated measures analysis. Both crude and adjusted models revealed a significant impact of treatment regimen on infant LAZ, with average Z-scores being 0.277 higher in the dolutegravir arm compared with the efavirenz arm in both models (crude model: *P* = 0.040, 95% CI: 0.013–0.541; adjusted model: *P* = 0.039, 95% CI: 0.014–0.540). No significant influence of treatment was found on infant WAZ, WLZ or HCZ in crude or adjusted models. Adjusted models had an improved fit for WAZ (AIC: 4189.47 vs. 4193.94), WLZ (AIC: 4984.59 vs. 4989.89) and LAZ (AIC: 4954.62 vs. 4971.57), but not for HCZ (AIC: 4136.26 vs. 4135.97).

**TABLE 2.** Outcomes of Crude and Adjusted Linear Mixed-effect Models of WAZ, WLZ, LAZ and HCZ

Model Variables	Crude Model				Adjusted Model			
	β Coefficient (SE)	95% CI	*P*-value	AIC	β Coefficient (SE)	95% CI	*P*-value	AIC
WAZ								
Intercept	−0.160 (0.096)	−0.349 to 0.029	0.096	4193.94	0.028 (0.119)	−0.207 to 0.263	0.815	4189.47
Regimen	0.087 (0.135)	−0.180 to 0.354	0.521		0.080 (0.134)	−0.184 to 0.344	0.550	
Study site	-	-	-		−0.348 (0.134)	−0.612 to −0.083	0.010	
WLZ								
Intercept	0.334 (0.085)	0.167 to 0.501	<0.001	4989.89	0.522 (0.107)	0.311 to 0.732	<0.001	4984.59
Regimen	−0.035 (0.120)	−0.272 to 0.201	0.768		−0.041 (0.118)	−0.274 to 0.192	0.730	
Study site	-	-	-		−0.333 (0.119)	−0.568 to −0.099	0.006	
LAZ								
Intercept	−0.768 (0.094)	−0.954 to −0.582	<0.001	4971.57	−3.051 (1.345)	−5.700 to −0.402	0.024	4954.62
Regimen	0.277 (0.134)	0.013 to 0.541	0.040		0.277 (0.133)	0.014 to 0.540	0.039	
Study site	-	-	-		−0.198 (0.135)	−0.463 to 0.067	0.142	
Maternal height	-	-	-		0.015 (0.008)	−0.001 to 0.032	0.074	
HCZ								
Intercept	0.505 (0.087)	0.333 to 0.676	<0.001	4135.97	0.600 (0.110)	0.384 to 0.816	<0.001	4136.26
Regimen	0.209 (0.124)	−0.034 to 0.453	0.091		0.207 (0.123)	−0.036 to 0.450	0.095	
Study site	-	-	-		−0.177 (0.124)	−0.421 to 0.066	0.153	

Adjusted models all include study site as a covariate. Additionally, the model on LAZ includes maternal height as a covariate. Regimen is coded as dolutegravir–efavirenz. *P*-values of fixed effects were determined using a Wald test.

## DISCUSSION

In this evaluation of growth trajectories over the first 72 weeks of life in infants exposed to either dolutegravir-based or efavirenz-based ART during late pregnancy and breastfeeding, infants with perinatal exposure to efavirenz-based ART exhibited an average LAZ that was 0.277 units lower than that in infants exposed to dolutegravir-based ART. The mean LAZ in the dolutegravir arm at 72 weeks was −0.92, compared with −1.52 in the efavirenz arm. Overall, no significant differences were found in average WAZ, WLZ and HCZ between infants with perinatal exposure to dolutegravir-based versus efavirenz-based ART.

In our population of HIV-exposed uninfected infants, mean WAZ remained close to WHO standards (WAZ = 0) in the first year of life but declined between weeks 48 and 72 in both study arms, consistent with previous research.^19^ Mean LAZ were near WHO standards at birth but decreased substantially over the first 72 weeks of life in both study arms, consistent with previous findings in this population.^19,20^ The observed declines in WAZ and LAZ in both study arms were not ART-specific and may be caused by various mechanisms, including in utero infections (other than HIV), indirect effects of in utero exposure to HIV and ART (eg, maternal immune activation or nutritional compromise), food insecurity, a high burden of endemic infectious diseases, and/or potential socioeconomic issues in HIV-affected households.^21^

The impact of treatment regimen on infant growth was analyzed using crude and adjusted models. Previous studies have shown that country and maternal height influence infant growth based on differences in food security, genetics, parental education and employment, breastfeeding practices, and health care access between countries.^17,18^ The impact of maternal height on infant growth is largely attributed to genetic factors.^17^ Given the well-documented and plausible influence of country and maternal height on infant growth, adjusting for these factors can provide a more refined understanding of the treatment effect and be applied when it improved model fit.

Consistent with the findings of the VESTED trial, our results showed small nonsignificant differences in WLZ between the dolutegravir arm and the efavirenz arm and higher LAZ in the dolutegravir arm compared with the efavirenz arm. In contrast to the VESTED trial, we observed no significant difference in WAZ between study arms.^9^ Differences in infant growth outcomes between treatments may be explained by several factors. First, a dolutegravir-based regimen achieves a faster reduction in viral load than an efavirenz-based regimen, resulting in reduced infant HIV-exposure and improved maternal health.^4^ Second, postnatal dolutegravir exposure has been associated with weight gain in children.^8^ The mechanisms behind this phenomenon remain uncertain, but it is plausible that perinatal exposure to dolutegravir may also affect infant growth. On top of this, perinatal exposure to efavirenz is linked to more severe rates of stunting compared with perinatal exposure to dolutegravir, indicating that perinatal efavirenz exposure could impair infant growth.^10^ Third, the transfer of dolutegravir and efavirenz across the placenta and into breastmilk differs, and because of this, dolutegravir causes higher in utero exposure but lower postpartum exposure than efavirenz.^22–24^ While increased perinatal ART exposure may protect against HIV transmission, it could also elevate the risk of adverse effects, including impaired infant growth. Finally, the median duration of exclusive breastfeeding was higher in infants in the dolutegravir arm compared with those in the efavirenz arm (3 months vs. 1 month), which may have positively impacted growth outcomes, as breastfeeding supports optimal infant growth by providing essential nutrients, promoting healthy immune development, and fostering overall growth and development.^25^

Discrepancies between our findings and the outcomes of the VESTED trial may partially be explained by the timing of ART initiation, as mothers in the VESTED trial-initiated treatment in the second trimester, whereas those in the DolPHIN-2 trial started in the third trimester. However, the impact of the timing of ART initiation on infant growth was not investigated in this study, and prior studies show inconsistent findings.^26,27^ Another reason for these discrepancies may be the difference in methodology. A major strength of our study is the use of linear mixed-effect models that account for repeated measures over time. The VESTED trial used 2-sample *t*-tests for the comparisons of mean Z-scores between treatment arms, not accounting for the correlation between repeated measurements.

Limitations of the study include the relatively small sample size (n = 232), as well as the use of Fenton and WHO growth charts rather than local or population-specific ones that might have been more appropriate for our study population. For preterm infants, WAZ, LAZ and HCZ after 50 weeks of postmenstrual age, as well as WLZ from birth onwards, were calculated using WHO growth charts due to the unavailability of Fenton charts for these outcomes during these time frames. However, since WHO charts do not account for prematurity, this probably resulted in an underestimation of the actual growth patterns in preterm infants. Due to incomplete attendance at each study visit, growth measurements were not attained for all participants at every visit. However, linear mixed-effect models can address this issue by assuming that missing data occurs at random. As gestational age was estimated in the third trimester, outcomes contained some uncertainty. Therefore, infant ages were not corrected for gestational age at delivery.

This study evaluated the effects of ART exposure during late pregnancy and breastfeeding on infant growth over a longer period than previous research. However, extended follow-up is needed to assess long-term growth outcomes, as the first 1000 days are critical for development.^28^ Additionally, further research on the effects of perinatal exposure to other ART regimens on infant growth will be warranted if new treatment options emerge.^18^ Ongoing safety surveillance and use of registries are crucial to monitor the impact of other antiretroviral drugs during pregnancy and to ensure optimal health outcomes for this important growing population, particularly given challenges with gestational dating and delayed identification of adverse events. Our findings of improved infant growth outcomes with a dolutegravir-based regimen compared with an efavirenz-based regimen support the WHO guidelines in recommending a dolutegravir-based regimen over an efavirenz-based regimen for the first- and second-line treatment of pregnant women living with HIV.

## Supplementary Material



## References

[1] World Health Organization. Consolidated guidelines on the use of antiretroviral drugs for treating and preventing HIV infection: recommendations for a public health approach. 2nd ed. June 1, 2016. Available at: https://www.who.int/publications/i/item/9789241549684. Accessed June 10, 2024. 27466667

[2] Evans C Jones CE Prendergast AJ. HIV-exposed, uninfected infants: new global challenges in the era of paediatric HIV elimination. Lancet Infect Dis. 2016;16:e92–e107. 27049574 10.1016/S1473-3099(16)00055-4

[3] World Health Organization. Updated recommendations on first-line and second-line antiretroviral regimens and post-exposure prophylaxis and recommendations on early infant diagnosis of HIV. January 1, 2018. Available at: https://www.who.int/publications/i/item/WHO-CDS-HIV-18.51. Accessed June 10, 2024.

[4] Kintu K Malaba TR Nakibuka J; DolPHIN-2 Study Group. Dolutegravir versus efavirenz in women starting HIV therapy in late pregnancy (DolPHIN-2): an open-label, randomised controlled trial. Lancet HIV. 2020;7:e332–e339. 32386721 10.1016/S2352-3018(20)30050-3 PMC10877544

[5] Powis KM Smeaton L Hughes MD. In-utero triple antiretroviral exposure associated with decreased growth among HIV-exposed uninfected infants in Botswana. AIDS. 2016;30:211–220. 26684818 10.1097/QAD.0000000000000895 PMC4685731

[6] Ramokolo V Goga AE Lombard C. In Utero ART exposure and birth and early growth outcomes among HIV-exposed uninfected infants attending immunization services: results from National PMTCT Surveillance, South Africa. Open Forum Infect Dis. 2017;4:ofx187. 29062860 10.1093/ofid/ofx187 PMC5641411

[7] Rosala-Hallas A Bartlett JW Filteau S. Growth of HIV-exposed uninfected, compared with HIV-unexposed, Zambian children: a longitudinal analysis from infancy to school age. BMC Pediatr. 2017;17:80. 28302082 10.1186/s12887-017-0828-6 PMC5356250

[8] Zash R Jacobson DL Diseko M. Comparative safety of antiretroviral treatment regimens in pregnancy. JAMA Pediatr. 2017;171:e172222. 28783807 10.1001/jamapediatrics.2017.2222 PMC5726309

[9] Koay WLA Dirajlal-Fargo S Levy ME; DC Cohort Executive Committee. Integrase strand transfer inhibitors and weight gain in children and youth with perinatal human immunodeficiency virus in the DC Cohort. Open Forum Infect Dis. 2021;8:ofab308. 34295943 10.1093/ofid/ofab308 PMC8291625

[10] Lynda Stranix-Chibanda for the IMPAACT 2010 protocol team and investigators University of Zimbabwe Clinical Trials Research Centre Harare Z. Growth of infants with perinatal exposure to maternal DTG vs EFV and TDF vs TAF: the randomised IMPAACT 2010 trial.

[11] Sudfeld CR McCoy DC Danaei G. Linear growth and child development in low- and middle-income countries: a meta-analysis. Pediatrics. 2015;135:e1266–e1275. 25847806 10.1542/peds.2014-3111

[12] Ebonwu J Mumbauer A Uys M. Determinants of late antenatal care presentation in rural and peri-urban communities in South Africa: a cross-sectional study. PLoS One. 2018;13:e0191903. 29518082 10.1371/journal.pone.0191903 PMC5843210

[13] Chou JH Roumiantsev S Singh R. PediTools electronic growth chart calculators: applications in clinical care, research, and quality improvement. J Med Internet Res. 2020;22:e16204. 32012066 10.2196/16204 PMC7058170

[14] World Health Organization. WHO Anthro Survey Analyser: Quick Guide. Geneva, Switzerland; 2019.

[15] Townsend R Khalil A. Fetal growth restriction in twins. Best Pract Res Clin Obstet Gynaecol. 2018;49:79–88. 29661565 10.1016/j.bpobgyn.2018.02.004

[16] Omoni AO Ntozini R Evans C. Child growth according to maternal and child HIV status in Zimbabwe. Pediatr Infect Dis J. 2017;36:869–876. 28198792 10.1097/INF.0000000000001574 PMC5571879

[17] Addo OY Stein AD Fall CH; Consortium on Health Orientated Research in Transitional Societies (COHORTS) Group. Maternal height and child growth patterns. J Pediatr. 2013;163:549–554. 23477997 10.1016/j.jpeds.2013.02.002 PMC3711792

[18] Nyemba DC Kalk E Vinikoor MJ. Growth patterns of infants with in-utero HIV and ARV exposure in Cape Town, South Africa and Lusaka, Zambia. BMC Public Health. 2022;22:55. 35000577 10.1186/s12889-021-12476-z PMC8744341

[19] McHenry MS Apondi E Ayaya SO. Growth of young HIV-infected and HIV-exposed children in western Kenya: a retrospective chart review. PLoS One. 2019;14:e0224295. 31800588 10.1371/journal.pone.0224295 PMC6892498

[20] Evans C Chasekwa B Ntozini R; Sanitation Hygiene Infant Nutrition Efficacy (SHINE) Trial Team. Mortality, human immunodeficiency virus (HIV) transmission, and growth in children exposed to HIV in Rural Zimbabwe. Clin Infect Dis. 2021;72:586–594. 31974572 10.1093/cid/ciaa076 PMC7884806

[21] Wedderburn CJ Evans C Yeung S. Growth and neurodevelopment of HIV-exposed uninfected children: a conceptual framework. Curr HIV/AIDS Rep. 2019;16:501–513. 31732866 10.1007/s11904-019-00459-0 PMC6920255

[22] Waitt C Orrell C Walimbwa S. Safety and pharmacokinetics of dolutegravir in pregnant mothers with HIV infection and their neonates: a randomised trial (DolPHIN-1 study). PLoS Med. 2019;16:e1002895. 31539371 10.1371/journal.pmed.1002895 PMC6754125

[23] Cressey TR Stek A Capparelli E; IMPAACT P1026s Team. Efavirenz pharmacokinetics during the third trimester of pregnancy and postpartum. J Acquir Immune Defic Syndr. 2012;59:245–252. 22083071 10.1097/QAI.0b013e31823ff052 PMC3288559

[24] Schneider S Peltier A Gras A. Efavirenz in human breast milk, mothers’, and newborns’ plasma. J Acquir Immune Defic Syndr. 2008;48:450–454. 18614925 10.1097/QAI.0b013e31817bbc21

[25] Wallenborn JT Levine GA Carreira Dos Santos A. Breastfeeding, physical growth, and cognitive development. Pediatrics. 2021;147:e2020008029. 33888567 10.1542/peds.2020-008029

[26] Ejigu Y Magnus JH Sundby J. Differences in growth of HIV-exposed uninfected infants in ethiopia according to timing of in-utero antiretroviral therapy exposure. Pediatr Infect Dis J. 2020;39:730–736. 32516280 10.1097/INF.0000000000002678 PMC7360102

[27] M le Roux S Jao J Brittain K. Tenofovir exposure in utero and linear growth in HIV-exposed, uninfected infants. AIDS. 2017;31:97–104. 27898591 10.1097/QAD.0000000000001302 PMC5814299

[28] Black RE Victora CG Walker SP; Maternal and Child Nutrition Study Group. Maternal and child undernutrition and overweight in low-income and middle-income countries. Lancet. 2013;382:427–451. 23746772 10.1016/S0140-6736(13)60937-X

