# Supplementary material for: The Effects on the Growth of HIV-exposed Uninfected Infants of Initiating Dolutegravir-based Versus Efavirenz-based cART in Late Pregnancy (DolPHIN-2)
Source: Pediatr Infect Dis J. 2025 Jul 18;44(11):1066–71. doi: 10.1097/INF.0000000000004902 (PMC12506683; doi:10.1097/INF.0000000000004902)
Supplement: Supplementary file 1 [file inf-44-1066-s001.pdf]

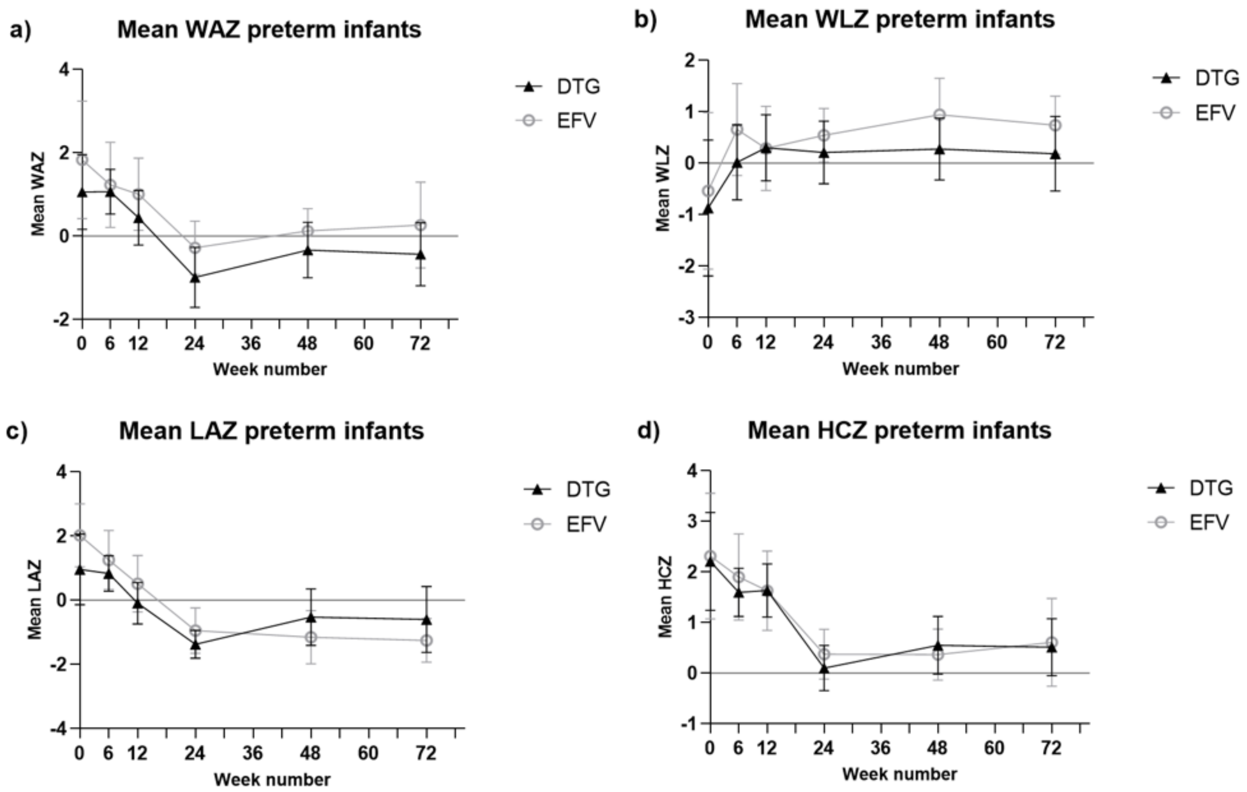

Supplemental Digital Content 1: Mean weight-for age Z-scores (WAZ) (a), weight-for-length Z-scores (WLZ) (b), length-for-age Z-scores (LAZ) (c), and head circumference-for-age Z-scores (HCZ) (d) of premature infants, with 95% confidence intervals, over the first 72 weeks of life. Results are stratified by treatment: dolutegravir (DTG) (filled triangle) or efavirenz (EFV) (open circle). Means are depicted at study visits around weeks 0, 6 (± 2 weeks), 12 (± 2 weeks), 24 (± 4 weeks), 48 (± 4 weeks), and 72 (± 4 weeks). Results outside of these intervals are excluded from the figure.
