# Supplementary material for: The Effects on the Growth of HIV-exposed Uninfected Infants of Initiating Dolutegravir-based Versus Efavirenz-based cART in Late Pregnancy (DolPHIN-2)
Source: Pediatr Infect Dis J. 2025 Jul 18;44(11):1066–71. doi: 10.1097/INF.0000000000004902 (PMC12506683; doi:10.1097/INF.0000000000004902)
Supplement: Supplementary file 3 [file inf-44-1066-s003.pdf]

Supplemental Digital Content 3: Outcomes of linear mixed-effect models of WAZ, WLZ, LAZ, and HCZ with continuous slope

	Model variables	β coefficient (SE)	95% CI	P-value
WAZ	Intercept	0.023 (0.102)	-0.178 to 0.224	0.822
	Regimen	0.087 (0.144)	-0.196 to 0.371	0.554
	Time(continuous)	-0.007 (0.001)	-0.009 to -0.004	<0.001
	Regimen*Time(continuous)	0.000 (0.002)	-0.004 to 0.003	0.810
WLZ	Intercept	0.348 (0.097)	0.158 to 0.539	<0.001
	Regimen	-0.135 (0.137)	-0.406 to 0.135	0.326
	Time(continuous)	-0.001 (0.002)	-0.004 to 0.003	0.765
	Regimen*Time(continuous)	0.004 (0.002)	-0.001 to 0.009	0.125
LAZ	Intercept	-0.303 (0.102)	-0.503 to -0.103	0.003
	Regimen	0.216 (0.144)	-0.067 to 0.499	0.134
	Time(continuous)	-0.017 (0.001)	-0.020 to -0.014	<0.001
	Regimen*Time(continuous)	0.001 (0.002)	-0.002 to 0.005	0.480
HCZ	Intercept	0.578 (0.093)	0.395 to 0.761	<0.001
	Regimen	0.273 (0.132)	0.014 to 0.532	0.039
	Time(continuous)	-0.003 (0.001)	-0.005 to 0.000	0.022
	Regimen*Time(continuous)	-0.003 (0.002)	-0.006 to 0.001	0.120

Regimen is coded as dolutegravir – efavirenz
P-values of fixed effects were determined using a Wald test
