# Supplementary material for: The Effects on the Growth of HIV-exposed Uninfected Infants of Initiating Dolutegravir-based Versus Efavirenz-based cART in Late Pregnancy (DolPHIN-2)
Source: Pediatr Infect Dis J. 2025 Jul 18;44(11):1066–71. doi: 10.1097/INF.0000000000004902 (PMC12506683; doi:10.1097/INF.0000000000004902)
Supplement: Supplementary file 4 [file inf-44-1066-s004.pdf]

Supplemental Digital Content 4: Outcomes of linear mixed-effect models of WAZ, WLZ, LAZ, and HCZ with categorical slope

	Model variables	β coefficient (SE)	95% CI	P-value
WAZ	Intercept	-0.418 (0.131)	-0.674 to -0.161	0.001
	Regimen	0.024 (0.190)	-0.349 to 0.398	0.898
	Time(categorical)			
	Weeks 0-6	0.231 (0.146)	-0.055 to 0.516	0.113
	Weeks 6-12	0.487 (0.130)	0.222 to 0.733	<0.001
	Weeks 12-24	0.513 (0.130)	0.257 to 0.768	<0.001
	Weeks 24-48	0.248 (0.131)	-0.009 to 0.505	0.059
	Weeks 48-72	0.182 (0.132)	-0.077 to 0.442	0.168
	Regimen*Time(categorical)			
	Weeks 0-6	0.233 (0.211)	-0.181 to 0.646	0.269
	Weeks 6-12	0.096 (0.189)	-0.275 to 0.467	0.612
	Weeks 12-24	-0.017 (0.189)	-0.388 to 0.354	0.929
	Weeks 24-48	-0.042 (0.192)	-0.419 to 0.334	0.826
	Weeks 48-72	0.127 (0.194)	-0.253 to 0.507	0.511
WLZ	Intercept	0.246 (0.146)	-0.041 to 0.534	0.093
	Regimen	-0.031 (0.216)	-0.455 to 0.392	0.885
	Time(categorical)			
	Weeks 0-6	-0.507 (0.187)	-0.874 to -0.140	0.007
	Weeks 6-12	0.337 (0.166)	0.011 to 0.662	0.043
	Weeks 12-24	0.369 (0.168)	0.039 to 0.699	0.028
	Weeks 24-48	0.259 (0.168)	-0.072 to 0.589	0.125
	Weeks 48-72	0.162 (0.170)	-0.172 to 0.496	0.341
	Regimen*Time(categorical)			
	Weeks 0-6	0.022 (0.270)	-0.508 to 0.553	0.934
	Weeks 6-12	-0.245 (0.243)	-0.722 to 0.233	0.315
	Weeks 12-24	-0.108 (0.244)	-0.587 to 0.271	0.658
	Weeks 24-48	-0.035 (0.248)	-0.522 to 0.452	0.887
	Weeks 48-72	0.132 (0.249)	-0.357 to 0.621	0.597
LAZ	Intercept	-1.434 (0.135)	-1.700 to -1.168	<0.001
	Regimen	0.313 (0.198)	-0.075 to 0.702	0.113
	Time(categorical)			
	Weeks 0-6	1.265 (0.153)	0.965 to 1.566	<0.001
	Weeks 6-12	1.105 (0.136)	0.838 to 1.372	<0.001
	Weeks 12-24	0.855 (0.138)	0.585 to 1.125	<0.001
	Weeks 24-48	0.648 (0.138)	0.376 to 0.918	<0.001
	Weeks 48-72	0.478 (0.139)	0.204 to 0.751	<0.001
	Regimen*Time(categorical)			
	Weeks 0-6	-0.077 (0.222)	-0.513 to 0.358	0.727
	Weeks 6-12	-0.021 (0.199)	-0.412 to 0.370	0.916
	Weeks 12-24	-0.139 (0.200)	-0.531 to 0.252	0.485
	Weeks 24-48	-0.155 (0.203)	-0.513 to 0.283	0.570
	Weeks 48-72	-0.139 (0.204)	-0.540 to 0.261	0.494
HCZ	Intercept	0.441 (0.122)	0.202 to 0.680	<0.001
	Regimen	0.099 (0.177)	-0.248 to 0.445	0.577
	Time(categorical)			
	Weeks 0-6	-0.055 (0.137)	-0.323 to 0.213	0.688
	Weeks 6-12	0.137 (0.122)	-0.103 to 0.377	0.263
	Weeks 12-24	0.290 (0.123)	0.049 to 0.531	0.018
	Weeks 24-48	0.122 (0.123)	-0.199 to 0.363	0.319
	Weeks 48-72	-0.004 (0.124)	-0.248 to 0.239	0.971
	Regimen*Time(categorical)			
	Weeks 0-6	0.354 (0.197)	-0.032 to 0.741	0.072
	Weeks 6-12	0.156 (0.177)	-0.191 to 0.503	0.376
	Weeks 12-24	0.124 (0.177)	-0.223 to 0.471	0.483
	Weeks 24-48	-0.035 (0.179)	-0.387 to 0.317	0.846
	Weeks 48-72	0.015 (0.182)	-0.341 to 0.372	0.932

Regimen is coded as dolutegravir – efavirenz
P-values of fixed effects were determined using a Wald test
